# Disruption of human plasma cell differentiation by an environmental polycyclic aromatic hydrocarbon: a mechanistic immunotoxicological study

**DOI:** 10.1186/1476-069X-9-15

**Published:** 2010-03-24

**Authors:** Lenka L Allan, David H Sherr

**Affiliations:** 1 Department of Environmental Health, Boston University School of Public Health, Boston, MA, 02118, USA; 2 Department of Pathology and Laboratory Medicine, University of British Columbia, Vancouver, British Columbia, Canada

## Abstract

**Background:**

The AhR is a ligand-activated transcription factor that mediates immunosuppression induced by environmental PAH and HAH. Recently, a critical role for the AhR in development of T cells involved in autoimmunity (Th17 and Treg) has been demonstrated, supporting the hypothesis that the AhR plays a key role in immune regulation both in the presence and absence of environmental ligands. Despite these results with T cells systems, little is known of the role that the AhR plays in B cell development. We have demonstrated that B cell activation with CD40 ligand, a stimulus that models adaptive immunity, induces AhR expression in primary human B cells, suggesting that activation may increase human B cell sensitivity to AhR ligands and that the AhR may play a role in B cell development.

**Methods:**

To test these possibilities, we developed an in vitro system in which activated human B cells expressing high AhR levels are induced to differentiate into plasma cells. Consequently, the effects of benzo [a]pyrene, a prototypic environmental AhR ligand, on plasma cell differentiation could be investigated and this chemical could be exploited essentially as drug probe to implicate the role of the AhR in plasma cell development.

**Results:**

A previously unattainable level of B cell differentiation into plasma cells (up to 45% conversion) was observed. Benzo [a]pyrene significantly suppressed that differentiation. γ-Irradiation after an initial proliferation phase induced by CD40 ligand and immediately prior to initiation of the differentiation phase blocked cell growth but did not affect cell viability or plasma cell differentiation. B [a]P suppressed differentiation whether or not cell growth was inhibited by γ-irradiation.

**Conclusions:**

1) Extensive proliferation is not required during the differentiation phase per se for CD40L-activated human B cells to undergo plasma cell differentiation, and 2) an environmental PAH blocks both proliferation and differentiation of AhR expressing B cells. The results uncover a new mechanism by which environmentally ubiquitous PAHs may negatively impact human B cell-mediated immunity.

## Background

Polycyclic aromatic hydrocarbons (PAHs) are ubiquitous environmental pollutants generated by the incomplete combustion of carbon sources. Numerous studies have demonstrated that a variety of PAHs are carcinogenic and immunosuppressive. Indeed, carcinogenic PAHs, such as benzo [a]pyrene (B [a]P), suppress both humoral (B cell-mediated) and cellular (T cell-mediated) immune responses. Most of these adverse effects are mediated by the aryl hydrocarbon receptor (AhR), a cytosolic receptor/transcription factor [1]. In some cases, lymphocyte immunotoxicity may be mediated indirectly through accessory cells. For example, PAH-induced pre-B cell apoptosis is mediated by AhR-expressing stromal cells in the bone-marrow microenvironment [2]. Direct effects of other PAHs on transformed B cells also have been reported [3]. Similarly, halogenated hydrocarbons impair the ability of thymic stroma to support developing T cell growth and/or differentiation [4-12].

Many studies that evaluate the mechanisms by which PAHs, or other related AhR ligands (e.g. halogenated aromatic hydrocarbons/HAHs), mediate immunosuppression have been performed in animal models. These studies reveal that AhR ligands suppress immunity by their ability to compromise virtually every stage of lymphocyte development, activation, and effector function studied. In this regard, PAHs and/or HAHs suppress mature B and T cell development in primary lymphoid organs [4-12] as well as inhibit antibody production, alloantigen-specific mixed-lymphocyte reactions, T cell cytokine production, effector and memory T cell development, cytotoxic T cell responses, and host resistance to infectious agents and transplantable tumors [7,13-19].

Perhaps most intriguing are recent studies demonstrating that the AhR is intimately involved in the development of T cell subsets, e.g., regulatory T cells (Treg) and IL-17-secreting Th17 cells, and that activation of the AhR in vivo enhances autoimmunity, presumably by up-regulating Th17 development and/or down-regulating Treg development [20-24]. This environmental chemical alteration in development of T cell subsets helps explain the outcomes of pioneering studies of Kerkvliet et al in which AhR activation was shown to suppress T cell-mediated tumor immunity or allograft rejection [25,26]. These results support our working hypothesis that the AhR plays a key role in immune regulation and that environmental AhR ligands can significantly compromise immunity by altering AhR-dependent lymphocyte development and/or function.

Despite these exciting results with T cells systems, little is known of the role that the AhR plays in B cell development in general and in human cells in particular. For example, while now classic studies demonstrated that low doses of TCDD (10^-9 ^M) suppress antibody secretion by immmortalized murine B cell lines [27,28], little is known of the ability of AhR ligands to affect human B cell differentiation into plasma cells, a critical event in the ultimate production of protective antibodies. The studies that have been performed in this area relied primarily on transformed cell lines or mixed lymphocyte populations with which it is difficult to pinpoint the cell subset directly affected by a given AhR ligand.

To begin to bridge this gap, we developed an in vitro model to demonstrate that activation of primary human B cells with CpG or CD40L, stimuli that mimic innate or adaptive B cell responses respectively, dramatically increases AhR expression [29]. These results suggest that B cell stimulation following pathogen exposure may increase B cell sensitivity to environmental AhR ligands through AhR up-regulation. Since CD40L-stimulated, B cells undergo rapid proliferation in germinal centers following antigenic stimulation and then differentiate into plasma cells, it seemed plausible that AhR ligands could interfere with the growth of what is presumed to be B cells expressing high AhR levels ("AhR^high^") and/or their differentiation into plasma cells. The former possibility was confirmed by the demonstration that B [a]P inhibits proliferation of CD40L-activated primary human B cells [29]. To test the latter possibility, we defined conditions under which purified human B cells could be induced to undergo differentiation in vitro and then evaluated the effects of B [a]P, a prototypical environmental PAH and AhR ligand, on that process. The results demonstrate that, while proliferation may be required for B cells to reach a state at which they are capable of differentiating into plasma cells, ongoing B cell proliferation is not required during the differentiation process itself. Furthermore, results suggest that an environmental PAH acts directly on AhR^high ^human B cells to suppress their differentiation into plasma cells, and that different classes of AhR ligands differentially affect biologic outcomes.

## Methods

### Chemicals

B [a]P (Sigma, St. Louis, MO) was dissolved in dimethylsulfoxide (DMSO) (Sigma). Cells were dosed from a 1000× stock so that the final concentration was 0.1%.

### Cell culture

CD40L-transfected L cells (American Type Tissue Culture Collection, VA) were maintained at 37°C in 10% CO_2 _in RPMI supplemented with 10% FBS, 2 mM L-glutamine, 5 μg/ml Plasmocin (Invivogen, San Diego, CA) and hypoxanthine thymidine (HT). Unless otherwise indicated, all culture reagents were obtained from Cellgro (Mediatech, Herndon, VA).

### B cell preparation

Peripheral blood mononuclear cells (PBMC) were prepared from anonymous individual blood donors (New York Biologics, Inc., New Jersey, NY) by centrifugation of 50-100 ml whole blood through Ficoll (Amersham Biosciences, Uppsala, Sweden) as previously described [29]. PBMCs were depleted of T cells by sheep red blood cell (ICN Biomedicals, Aurora, OH) rosetting and a second centrifugation through Ficoll. PBMCs were stained with FITC-labeled CD20-specific antibody (BD PharMingen (Chicago, IL) and purified by fluorescence-activated cell sorting (MoFlo, Dako Cytomation) based on forward and side scatter parameters and CD20 expression. Approximately 10^7 ^B cells (>99% CD20^+^) were recovered per donor from approximately 10^8 ^PBMCs.

### Plasma cell generation (two step cell culture)

In the first stage of plasma cell generation, purified B cells were plated at approximately 3 × 10^6 ^cells/well in 6-well plates on irradiated CD40L-transfected L cells in B cell media (Iscove's medium; Invitrogen, Carlsbad, CA) supplemented with 5% human AB serum (MP Biomedicals), 50 μg/ml transferrin (Invitrogen), 0.5% human serum albumin (Aventis Behring, Kanakakee, IL), 5 μg/ml insulin (Sigma), and 25 μg/ml Plasmocin plus the following cytokines: rIL-2 (50 U/ml), rIL-4 (50 ng/ml), rIL-10 (50 ng/ml) and rIL-12 (2 ng/ml). After 4 days of culture (step 2), B cells were harvested, washed and seeded in a single well (24 well plate) at 4 × 10^5^/ml media containing rIL-2 (50 U/ml), rIL-6 (25 ng/ml), rIL-10 (50 ng/ml), rIL-12 (2 ng/ml) and rIFN-α (100 U/ml) in the absence of CD40L-transfected L cells. All cytokines were obtained from Research Diagnostics Inc. (RDI, Flanders, NJ). The cell recovery after the second 4 day culture was over 2 × 10^6 ^cells/well, representing approximately a 6-fold increase in cell numbers during the second culture.

Cells were treated with vehicle (DMSO) or 10^-6 ^M B [a]P dissolved in DMSO on the first day of culture and/or on day 4 at the start of the second culture step. For all studies, cells were washed extensively after the first culture and the same number of viable cells, 4 × 10^5^, was added to each well in the second (differentiation) culture. In studies evaluating the requirement for cell proliferation during plasma cell differentiation, activated B cells were γ-irradiated (700 Rads) on day 4 prior to the initiation of the second culture. Where indicated, 30 μM ZVAD-fmk (Biomol International, Plymouth Meeting, PA) was added during the second culture step to maximize cell viability. Approximately 4 × 10^5 ^cells/ml were recovered in vehicle-treated wells following cell irradiation with or without ZVAD-fmk support.

### Surface phenotype and viability of cultured B and plasma cells

Cells were harvested on day 8 of culture and viability was determined by trypan blue and propidium iodide exclusion by light microscopy and flow cytometry, respectively. Phenotypic analyses of B and plasma cells were performed using FITC-conjugated anti-CD20, PE-conjugated anti-CD38 antibody (BD PharMingen (Chicago, IL) or fluorochrome-labeled isotype controls. Non-specific mAb binding was blocked by incubating cells for 10 min in PBS containing 5% FBS, 1% sodium azide and 0.01 mg/ml normal mouse IgG (Caltag Laboratories, Burlingame, CA). Cells then were labeled with mAb or isotype matched control antibodies according to the manufacturer's instructions. Following one wash, cells were fixed in PBS containing 3.7% paraformaldehyde and analyzed in a Becton Dickinson FACScan flow cytometer using CellQuest software (BD Biosciences). Quadrants were set using dot plots obtained with isotype controls.

### Giemsa stain

Following the second culture, sorted CD20^lo^/CD38^hi ^cells were resuspended in PBS (70,000 cells/100 μl) and cytocentrifuged (Thermo Shandon, Thermo Electron Corporation, Pittsburg, PA) for 5 minutes at 400 rpm onto pre-cleaned Colorfrost/Plus microscope slides (Fisher Scientific). Slides were immediately fixed with 90% ethanol for 1 minute and allowed to dry before staining for 30 minutes with 1:4 Giemsa stain/deionized water solution (Sigma). Excess stain was removed from slides by dipping in water, followed by dipping in 0.01% acetic acid. Slides were dehydrated by dipping into 95% ethanol and 100% ethanol, placed into xylene and mounted with Permount (Fisher Scientific) and a coverslip. Images were digitally captured using DIC optics (Nikon).

### Proliferation assays

CD40L-activated B cells were plated at a density of 10^5 ^cells/well into 96-well plates for 20 hours. [^3^H]-thymidine (1 μCi/well) was added and plates were incubated for an additional 18 hours. Cells were harvested onto filter strips using a cell harvester (Brandel, Gaithersbug, MD) and radionucleotide incorporation was measured using a liquid scintillation counter (Wallac, Turku, Finland). For each donor, B cell treatments were performed in triplicate. The means of the triplicate radioactivity counts per minute (cpm) from each donor were used to obtain an average for each indicated data point.

### Apoptosis assay

Primary cultured B cells were harvested and washed once with cold PBS containing 5% FBS and 0.01 M sodium azide (Sigma). For propidium iodide (PI) staining, cells were resuspended in 0.15 ml hypotonic buffer containing 50 μg/ml PI (Sigma), 0.1% sodium citrate and 0.1% Triton X-100 and analyzed by flow cytometry as we previously described [1,29-31]. Cells undergoing DNA fragmentation (i.e. apoptosis) have a weaker PI fluorescence than those in the typical G_0_/G_1 _stages of cell cycle [31].

### Statistical Analyses

The Student's paired t-test and one-factor ANOVA were used to analyze the data using Statview (SAS Institute, Cary, NC). For ANOVA, the Dunnett's or the Tukey/Kramer multiple comparisons tests were used to compare experimental and vehicle-treated groups or to compare all possible combinations of groups respectively.

## Results

### Generation of plasma cells using two-step cell cultures

In order to address whether environmental AhR ligands affect the differentiation of human B cells into plasma cells, it was necessary first to develop an in vitro differentiation system in which significant numbers of human plasma cells could be reliably generated from activated, AhR^high ^B cells. To this end, modifications were made to previously published protocols in which activation of human B cells was shown to significantly up-regulate AhR expression [32,33]. In the first phase of the protocol, CD20^+ ^human B cells, purified from PBMC by fluorescence-activated cell sorting, were activated by growth on monolayers of human CD40L-transfected L cells for 4 days in the presence of rIL-2, rIL-4, rIL-10 and rIL-12.

**Figure 1:**
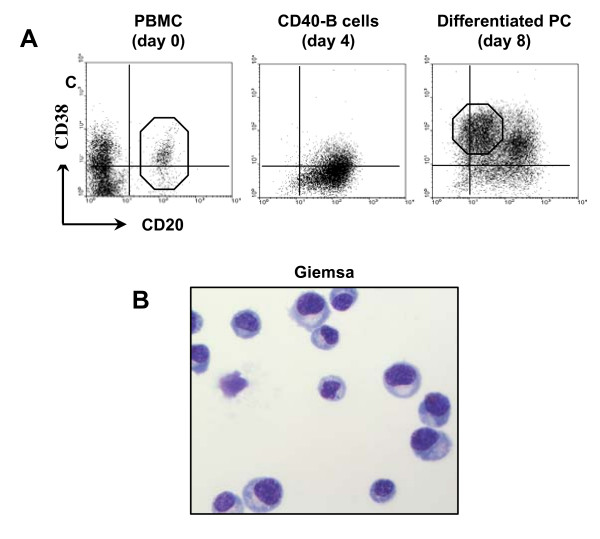
**Differentiation of peripheral human B cells into plasma cells**. CD20^hi ^B cells were purified by fluorescence-activated cell sorting and activated for 4 days on CD40L-expressing cells in the presence of rIL-2, rIL-4, rIL-10, and rIL-12. Activated B cells then were adjusted to 4.0 × 10^5 ^cells/ml and cultured for an additional 4 days without CD40L cells but with rIL-2, rIL-6, rIL-10, rIL-12 and rIFN-α. **A) **The phenotype of fresh PBMCs prior to sorting, and of B lineage cells obtained after the first and second cultures was determined by flow cytometry following staining with CD20- and CD38-specific or isotype control monoclonal antibodies. Quadrants were set using isotype control antibodies. CD20^high^/CD38^low ^starting B cells and CD20^lo^/CD38^hi ^plasma cells are indicated by the octagons in the leftmost and rightmost dot plots respectively. **B) **CD20^lo^/CD38^hi ^cells generated after the second culture were sorted by flow cytometry, cytocentrifuged onto glass slides, and treated with Giemsa stain to visualize plasma cell morphology.

Robust B cell proliferation was observed during the first culture period with B cell numbers increasing approximately 8-fold [29]. Significant up-regulation of AhR expression occurred within 24 hours of CD40L activation and remained high for several weeks [1,29]. After this first culture, the cells expressed high levels of CD20 and low levels of the plasma cell/activated lymphocyte marker CD38 (Figure 1A, center dot plot).

**Figure 2:**
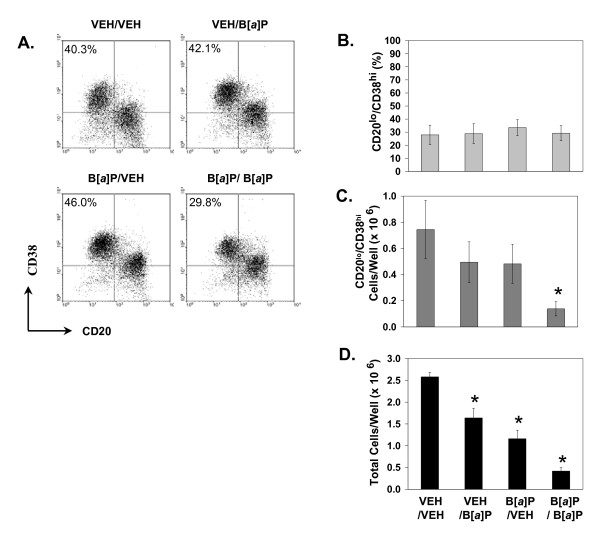
**B [*a*]P suppresses plasma cell production**. Purified CD20^+ ^B cells from 4 donors were individually cultured in two steps as in Figure 1 to generate plasma cells. Cultures were treated with vehicle or 10^-6 ^M B [*a*]P during the first (B [*a*]P/VEH), the second (VEH/B [*a*]P), or both (B [*a*]P/B [*a*]P) culture periods. **A) **Dot plots of cells obtained from a representative donor stained with CD20- and CD38-specific or isotype control antibodies are shown. Quadrants were set using isotype control antibodies. The percentages of CD20^lo^/CD38^hi ^plasma cells are indicated in the upper left quadrants. **B) **The percentages of CD20^lo^/CD38^hi ^plasma cells obtained after the second culture period when cells were exposed to vehicle or B [*a*]P during the first, second, or both cultures are presented as means + SE. **C) **The total number of CD20^lo^/CD38^hi ^plasma cells obtained after both cultures (day 8) are presented as means + SE. **D) **The total number of cells obtained after both cultures are presented as mean + SE. An asterisk (*) indicates a significant decrease relative to vehicle-treated cells (p < 0.05; Dunnett's).

Activated CD20^hi^/CD38^lo ^B cells then were cultured for an additional 4 days with rIL-2, rIL-10, rIL-12, rIL-6, and rIFN-α but without rIL-4 or CD40L-transfected cells. Although stimulation through CD40 ceased after the first culture of our system, B cell continued to divide during a second culture period. In control cultures from individual experiments using cells from five donors, the number of cells harvested at the end of the eight day culture period (2.6 × 10^6 ^± 0.1 cells/well; Figure 2D, first histogram) was approximately 6-fold higher than the number plated on day four, demonstrating a significant level of proliferation during the second culture. (Note that the number of cells entering the second culture was always adjusted to a constant number, i.e., 4.0 × 10^5 ^cells/well). Following this second culture step (culture day 8), 31 ± 17% (mean ± SE; n = 4 donors) of the cells expressed the phenotypic characteristics of plasma cells, i.e. low CD20 and high CD38 expression (Figure 1A, right dot plot).

Although many studies validate this phenotype as a hallmark of human antibody secreting plasma cells [32,33], CD20^lo^/CD38^hi ^cells induced in our two step culture were sorted by flow cytometry to >95% purity and stained with Giemsa to visualize cell morphology and to formally confirm their identity as plasma cells. As expected, these cells displayed morphologic characteristics of plasma cells, namely a basophilic cytoplasm, an accentric nucleus, and a pale Golgi zone (Figure 1B). It was concluded, therefore, that the 2-step culture was suitable for analysis of plasma cell development in the presence of environmental chemicals.

### B [a]P suppresses production of plasma cells

To determine the effect of B [a]P exposure on the production of plasma cells, B cells were treated with vehicle or 10^-6 ^M B [a]P during the first and/or second culture step and the number of CD20^lo^/CD38^hi ^plasma cells was assessed. Although individual experiments occasionally suggested a decrease in the percentage of CD20^lo^/CD38^hi ^plasma cells after treatment with B [a]P during both cultures (e.g., Figure 2A, lower right dot plot), no significant differences were observed overall in the percentage of plasma cells generated in cultures exposed to B [a]P during any point of the cultures (Figure 2B). Treatment with B [a]P during either the first or the second culture tended to decrease the absolute number of plasma cells recovered, although statistical significance (p < 0.05) was not reached unless B [a]P was present in both cultures (Figure 2C). The apparent decrease in plasma cell yield following B [a]P exposure coincided with a significant decrease in the total number of cells in cultures treated during the first, second, or both cultures (Figure 2D). B [a]P did not induce significant levels of overt death or apoptosis, as assayed by trypan blue exclusion and propidium iodide staining and flow cytometry, under any of the conditions shown in Figure 2 (~7% apoptotic in both vehicle and B [a]P-treated cultures). These experiments indicate that B [a]P significantly reduces the total number of B cells and compromises the production of plasma cells in this model. However, they do not distinguish between B [a]P-mediated effects on B cell proliferation during the second, differentiation culture, from proliferation-independent effects on B cell differentiation into plasma cells.

### B [a]P suppresses plasma cell differentiation during conditions of minimal proliferation

It has been proposed that differentiation of B cells into plasma cells requires cell proliferation, at least during an initial stage of activation during which activated B cells become differentiation-competent [34-39]. As noted above, CD40L-activated B cells proliferate during both the initial activation culture and the subsequent differentiation culture. Previously, we demonstrated that B [a]P inhibits proliferation of CD40L-activated B cells [34-39]. Consequently, the reduced recovery of plasma cells in B [a]P-treated cultures could reflect a reduction in cell proliferation required to generate differentiation-competent cells during the first or second culture and/or inhibition of differentiation itself in the second culture. To develop a model in which the effects of B [a]P on B cell proliferation could be dissociated from its putative effects on differentiation, purified human B cells were activated for the first four days with CD40 ligand with cytokines and then γ-irradiated (700 Rads) to block further cell proliferation.

**Figure 3:**
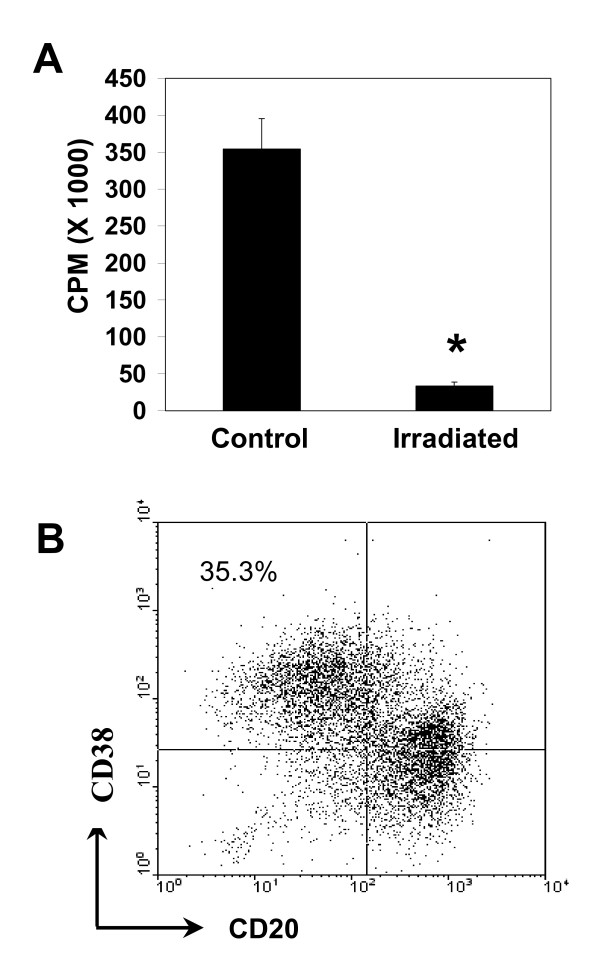
**γ-Irradiation inhibits cell growth but has no effect on B cell differentiation into plasma cells**. Purified CD20^+ ^B cells were activated for 4 days as described in Figure 1. Activated B cells were then γ-irradiated (700 Rads) and cultured without CD40L-transfected cells but with IL-2, IL-6, IL-10, IL-12 and IFN-α. **A**) [^3^H]-thymidine was added to cultures of irradiated and control cells at 21 hours and [^3^H]-thymidine incorporation assayed 18 hours later. Data from 3 donors are presented as mean cpm ± SE. An asterisk indicates a significant decrease in [^3^H]-thymidine incorporation as compared with corresponding non-irradiated controls (p < 0.05; paired t-test). **B) **Cells cultured as in "A" above were phenotyped for CD20 and CD38 expression by flow cytometry 4 days after irradiation and culture under plasma cell differentiation conditions. Dot plots obtained with cells from a representative donor are shown. The percentage of CD20^lo^/CD38^hi ^plasma cells is indicated in the upper left quadrant.

As expected, little or no growth was observed in the irradiated B cells, as indicated by the minimal level of [^3^H]-thymidine incorporation during an 18 hour period following irradiation (Figure 3A). When irradiated B cells were re-cultured under conditions that induce plasma cell differentiation, 41.8 ± 3.2% (mean ± SE, n = 4 donors) of the cells differentiated into CD20^lo^/CD38^hi ^plasma cells (e.g. Figure 3B). This level of plasma cell differentiation was comparable to that seen in non-irradiated cultures (Figure 2). These results demonstrate that inhibition of cell division during the second stage culture has no discernable effect on differentiation, a result consistent with the hypothesis that, after an initial proliferative phase, differentiation can occur in the absence of continual cell proliferation [33,40-43].

**Figure 4:**
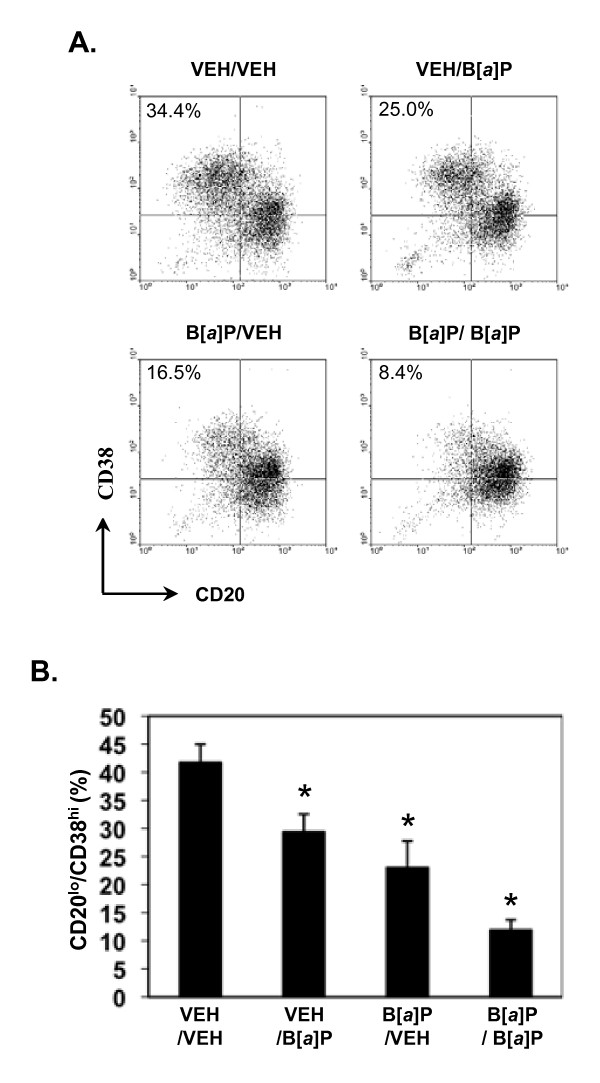
**B [*a*]P suppresses plasma cell differentiation under conditions of minimal proliferation**. Purified B cells were activated for 4 days as described in Figure 1. B cells then were γ-irradiated (700 Rads) and re-cultured for an additional 4 days under plasma cell differentiation conditions. Cells were treated with vehicle or 10^-6 ^M B [*a*]P during the first and/or second culture periods. **A) **Cells were phenotyped for CD20 and CD38 expression by flow cytometry. Dot plots obtained with cells from a representative donor (4 total) are shown. The percentages of CD20^lo^/CD38^hi ^plasma cells are indicated in the upper left quadrants. **B) **Data from cells obtained from four donors and treated as in "A" are presented as mean percentage of CD20^lo^/CD38^hi ^cells + SE. An asterisk (*) indicates a significant decrease relative to vehicle-treated cells (p < 0.05; Dunnett's).

Given these results, it was possible to determine the putative effects of B [a]P exposure on plasma cell differentiation during the second culture under conditions of minimal cellular proliferation. Treatment of activated, irradiated human B cells with B [a]P only during the differentiation phase (i.e., the second culture) significantly reduced the percentage (Figure 4A and 4B: VEH/B [a]P) and the absolute number (not shown) of CD20^lo^/CD38^hi ^plasma cells recovered. These results strongly suggest that B [a]P inhibits the process of plasma cell differentiation independent of its ability to suppress proliferation of activated B cells.

Similarly, treatment with B [a]P in the first culture step, during which extensive proliferation takes place, resulted in a significant reduction in the number (not shown) and percentage of plasma cells recovered (Figure 4A and 4B: B [a]P/VEH). B [a]P treatment during both cultures (B [a]P/B [a]P) resulted in the greatest decrease in the percentage of plasma cells recovered, a result consistent with an additive effect of B [a]P on the generation/proliferation of differentiation-competent B cells in the first culture and on their ability to differentiate into plasma cells in the second culture.

**Figure 5:**
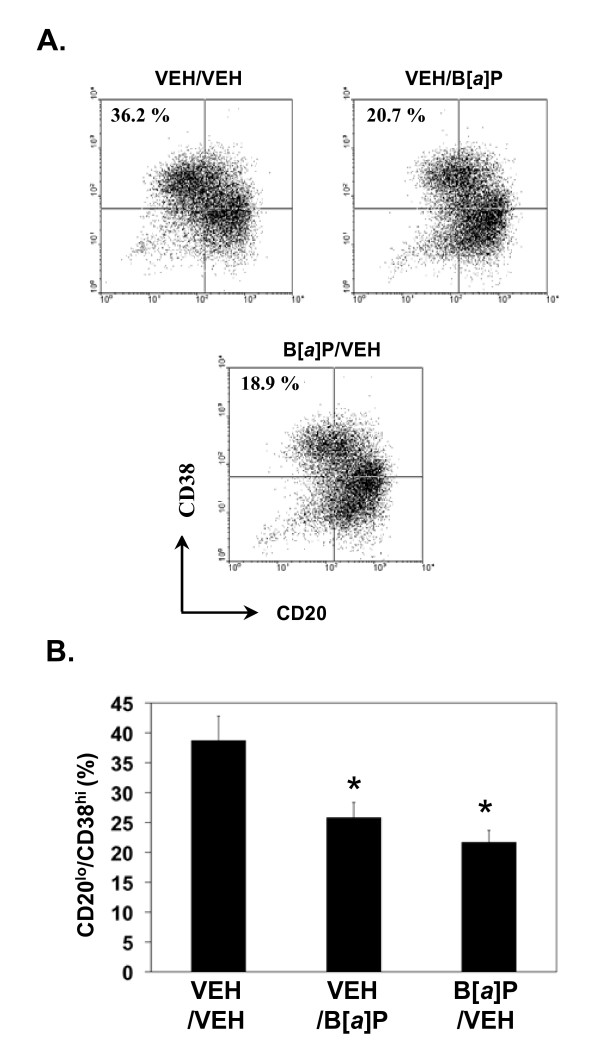
**Preferential plasma cell apoptosis does not account for a decrease in the percentage of plasma cells in B [*a*]P-treated cultures**. Purified B cells were activated for 4 days as described in Figure 1. B cells then were γ-irradiated and re-cultured in the presence of the pan-caspase inhibitor ZVAD-fmk (30 μM) for an additional 4 days. Cells were treated with vehicle or 10^-6 ^M B [*a*]P during the first and/or second culture periods. **A) **Cells were phenotyped for CD20 and CD38 expression by flow cytometry. Dot plots obtained with cells from a representative donor (4 total) are shown. The percentages of CD20^lo^/CD38^hi ^plasma cells are indicated in the upper left quadrants. **B) **The percentages of plasma cells (4 donors) obtained 4 days after irradiation (day 8) and after treatment with vehicle or 10^-6 ^M B [*a*]P in the presence or absence of 30 μM ZVAD-fmk are presented as means ± SE. An asterisk indicates a significant decrease in the percentage of plasma cells relative to control, vehicle-treated cells (p < 0.05; Dunnett's test).

Irradiated cells treated with vehicle or B [a]P appeared as healthy as non-irradiated cells at the end of culture as assessed by trypan blue and propidium iodide staining (~7% apoptotic). Nevertheless, it seemed formally possible that the apparent block by B [a]P of B cell differentiation could in part reflect preferential death of short-lived plasma cells [36] upon B [a]P exposure. To address this possibility, a separate series of experiments was performed in which a relatively high dose (30 μM) of a potent pan-caspase inhibitor, ZVAD-fmk [31], was added to the second culture to minimize B/plasma cell apoptosis. We have shown that >15 μM ZVAD-fmk or other caspase inhibitor efficiently blocks PAH-induced apoptosis of bone marrow B cell cells in vitro [31]. Apoptosis was measured by permeabilizing cells and staining DNA with propidium iodide, as described previously [31]. A relatively small population of cells (4.6 + 0.89%) was undergoing apoptosis following vehicle treatment (Table 1). No significant differences in the percentage of apoptotic cells were observed when cells were treated with B [a]P (Table 1). Nevertheless, B [a]P, added either at the beginning of the first culture period or during the differentiation stage, significantly decreased the percentage of CD20^lo^/CD38^hi ^plasma cells recovered (Figure 5A, B). These results are equivalent to those obtained with irradiated cells in the absence of ZVAD-fmk (Figure 4). Therefore, it is concluded that the decrease in plasma cell recovery seen after B [a]P treatment of the second, differentiation culture results primarily from a block in plasma cell differentiation and not from inhibition of growth or from overt toxicity manifest as preferential plasma cell death.

**Table 1:** B [*a*]P does not induce apoptosis during differentiation of irradiated B cells to plasma cells.

Treatment	% Apoptotic Cells
Vehicle/Vehicle	4.6 ± 0.9

Vehicle/B [*a*]P	6.5 ± 1.1

B [*a*]P/Vehicle	6.4 ± 0.8

Cells were activated with CD40L + rIL-2, rIL-4, rIL-10, and rIL-12 with vehicle or 10^-6 ^M B [a]P for 4 days as described in Materials and Methods. Cells were harvested, washed, irradiated (700 Rads) and re-plated at a concentration of 4 × 10^5^/ml in media containing rIL-2, rIL-6, rIL-10, rIL-12, and rIFN-α in the absence of CD40L-transfected L cells and in the presence of vehicle or B [a]P. The percentage of cells undergoing apoptosis on day 8 of culture was quantified by propidium iodide staining of permeabilized cells followed by flow cytometric analyses. Data are derived from samples shown in Figure 4 and are presented as means ± SE of cells falling into the sub G_0_/G_1 _peak as we previously described [30,31].

## Discussion

The adverse effects of PAH exposure on B cell-dependent, immune responses have been well documented [44-46]. While many studies performed in animal models demonstrated reduced immunoglobulin levels following in vivo or in vitro treatment with PAHs such as B [a]P or DMBA, the exact cellular mechanisms, e.g., inhibition of antigen presentation, reduction in T helper activity, or inhibition of antibody secretion, was generally not investigated and indeed could not be evaluated in the context of mixed lymphocyte populations. A significant advance came with the demonstration that an AhR ligand suppresses LPS-induced antibody secretion in a transformed murine B cell line in vitro [47,48]. While these studies demonstrated a mechanism through which an activated AhR could suppress immunoglobulin gene transcription, they did not address the effect of AhR ligands on the process of differentiation per se from activated B cell to plasma cell. The present study is the first to address specifically this issue and to extend studies to nontransformed, highly enriched primary human cells.

In addition, the current studies were motivated by our finding that human B cells, activated through CD40L as a surrogate for antigen-stimulated CD40L^+ ^T helper cells, up-regulate AhR expression within 24 hours by approximately 5-fold [29]. In addition to suggesting the possibility that the AhR plays an important role in B cell function, these results led us to investigate whether activated B cells undergo normal levels of proliferation and differentiation in the presence of AhR ligands.

Treatment of purified human B cells with B [a]P during either the first or second stage of culture resulted in a significant decrease in the total number of cells recovered (Figure 2C and 2D). This result alone indicates that suppression of humoral immunity may be mediated by direct effects of PAHs on activated AhR^high ^B cells. The maximal decrease in the number of plasma cells observed after B [a]P exposure during the entire culture period suggests an additive effect resulting from B [a]P inhibition of both B cell proliferation in the first culture and differentiation in the second.

In order to distinguish between the effects of B [a]P on B cell proliferation from additional effects on differentiation, we developed a system in which plasma cell differentiation could be studied under conditions of minimal cell proliferation. To this end, cells were γ-irradiated prior to the differentiation (second) culture. Prior to this stage, activated B cells do not express any markers characteristic of plasma cells. Radiation (700 R) significantly reduced proliferation without inducing apoptosis, as measured by propidium iodide staining or overt death as measured by trypan blue exclusion. Notably, the greater than 90% decrease in cell proliferation during the critical stage of differentiation, i.e., the second culture, had no effect on plasma cell differentiation. This important result extends previous reports in which it was concluded that extensive proliferation of B cells is required prior to differentiation into plasma cells [34-36,39]. In fact, the data presented herein indicate that, while CD40L^+ ^T cell-dependent proliferation of B cells prior to a differentiation signal may be required to generate "differentiation-competent" cells, little or no cell division is required during the differentiation phase itself [40-43,49].

To evaluate the effects of B [a]P on differentiation per se, B cells were irradiated at the beginning of the second culture to inhibit proliferation and then exposed to vehicle or B [a]P. B [a]P reduced the number (not shown) and percentage (Figure 4) of CD20^lo^/CD38^hi ^plasma cells significantly, thereby demonstrating for the first time that this prototypic PAH directly inhibits differentiation of B cells into plasma cells. It also was interesting to note that treatment of B cells during the first culture only, and prior to irradiation and differentiation, also decreased the percentage of plasma cells recovered despite the fact that cells were washed extensively to remove B [a]P and that cell numbers were adjusted to a standard level (4 × 10^5 ^cells/well) for the second culture. These results are consistent with the hypothesis that, by suppressing cell division, B [a]P exposure reduces the number of B cells that are competent to undergo differentiation, thereby negatively impacting the production of plasma cells.

While the mechanism underlying the block in B cell differentiation into plasma cells is not known, several possibilities exist. Pax5 is a transcription factor that promotes transcription of B cell lineage genes while repressing J-chain, IgH and XBP-1 genes, expression of which is necessary for plasma cell differentiation [49]. Notably, the murine and human Pax5 promoters contain three AhR response elements (AhREs), affording a direct transcriptional mechanism through which the AhR could up-regulate the expression of Pax5 and suppress genes required for plasma cell differentiation. In support of this possibility, at least one AhR ligand, TCDD, prevents an LPS-mediated decrease in Pax5 expression and suppresses antibody secretion in a transformed murine B cell line, CH12.LX [50].

A second mechanism through which AhR activation may alter the expression of genes required for plasma cell differentiation is through the modulation of Pax5 transcriptional activity. The Pax5 DNA recognition sequence (G/ANNCANTGNN**GCGT**/G**G**/AACC/GA/G) contains a consensus AhR 'core' binding site (bold) [51]. In fact, AhR found in nuclear extracts prepared from two human B cell lines recognizes a consensus Pax5 binding site in the CD19 promoter [52]. Therefore, it is possible that B [a]P augments repression of genes required for plasma cell differentiation, e.g. XBP-1, by virtue of their expression of Pax5/AhR binding sites.

The ability of B [a]P to suppresses plasma cell differentiation in the system described herein could reflect the ability of B cells to metabolize B [a]P. In this vein, we have shown that CYP1A1-dependent B [a]P metabolism into B [a]P-7,8 dihydrodiol-9,10-epoxide is required for suppression of CD40L-activated B cell proliferation [1]. Furthermore, B [a]P can be metabolized by cellular peroxidases to form B [a]P-quinones. These metabolites are converted into reactive oxygen species that induce oxidative stress. Cellular redox potential controls the DNA binding activity of several transcription factors, including NF-κB, AP-1, and p53 [53-57]. In addition, oxidative stress in human B cells induces expression of a multifunctional enzyme known as APE/Ref-1 (or HAP-1)[58]. APE/Ref-1 modulates the DNA binding activity of transcription factors such as Pax5 by reducing cysteine residues [59-62]. While oxidized Pax5 is unable to bind DNA, reduced Pax5 binds DNA regulatory sites with high affinity [58,61,62]. Thus, oxidative stress induced by B [a]P-quinones or B [a]P-epoxides may activate APE/Ref-1, which in turn promotes Pax5 transcriptional activity suppressing XBP-1 expression and plasma cell differentiation.

Regardless of the mechanism(s) through which B [a]P inhibits plasma cell differentiation, the data presented here demonstrate a second level, in addition to inhibition of B cell growth [1], through which this prototypic, environmental PAH is likely to suppress B cell responses. Collectively, the data support the hypothesis that up-regulation of AhR expression in activated human B cells renders them particularly susceptible to immunosuppressive PAHs.

## Conclusions

Using a novel human B cell differentiation system it was possible to evaluate the ability of environmental AhR ligands to directly suppress human B cell differentiation into plasma cells in the absence of other lymphoid cell populations. From these studies it is concluded that at least some AhR ligands are capable of suppressing the differentiation of activated human B cells into plasma cells without inducing cell death. Inhibition of differentiation with a prototypic PAH was seen even in the absence of B cell proliferation or significant levels of cell death, suggesting that a specific AhR-regulated signaling pathway(s) is impacted by PAH exposure. Taken with our previous studies showing up-regulation of AhR expression and activity in activated human B cells [29], these studies support the hypothesis that activated B cells, which express high levels of the AhR, are particularly susceptible to PAH exposure and that the AhR may play an important role in differentiation from activated B cell to plasma cell. These results suggest a mechanistic underpinning for epidemiological studies in which exposure to environmental chemicals, including metabolizable AhR ligands, results in reduced antibody responses [63].

## Abbreviations

AhR: Aryl Hydrocarbon Receptor; B [a]P: Benzo [a]pyrene; HAH: Halogenated Aromatic Hydrocarbon(s); PAH: Polycyclic Aromatic Hydrocarbon(s).

## Competing interests

The authors declare that they have no competing interests.

## Authors' contributions

LA and DS jointly conceived of the project, compiled and analyzed the data, and co-wrote the manuscript. LA developed the plasma cell differentiation assay and performed the experiments, data from which are presented herein. All authors have read and given final approval of the version to be published.
